# 2648. Taming the Cytokine Storm: Baricitinib versus Tocilizumab for COVID-19 Hyperinflammation

**DOI:** 10.1093/ofid/ofad500.2260

**Published:** 2023-11-27

**Authors:** Skyler Radziszewski, Siddharth Swamy, Ruchi Jain, Keri Bicking, Themba Nyirenda

**Affiliations:** Touro College of Pharmacy/Mount Sinai Hospital, New York, New York; Hackensack University Medical Center, Hackensack, New Jersey; Hackensack University Medical Center, Hackensack, New Jersey; Hackensack University Medical Center, Hackensack, New Jersey; HMHN Research Institute, Hackensack, New Jersey

## Abstract

**Background:**

Baricitinib and tocilizumab are potent immunomodulators that can reduce mortality in severe and critical COVID-19. The objective of this study was to compare the outcomes of patients with COVID-19 who were treated with either baricitinib or tocilizumab.

**Methods:**

This was a single-center, retrospective chart review. Patients were included if they were adults hospitalized with COVID-19 between January 2021 and October 2022 and received baricitinib or tocilizumab. Patients were excluded if they received both agents. Data collection included patient demographics and the predominant variant circulating at time of admission. The primary outcome was the percentage of patients with clinical improvement at day 14 after immunomodulator initiation. Clinical improvement was defined as a two point improvement on a six-point ordinal scale or hospital discharge. Secondary outcomes included clinical improvement at days 7 and 28 after immunomodulator initiation, progression to mechanical ventilation or ICU care, in-hospital mortality, and incidence of venous thromboembolism (VTE).

**Results:**

Four hundred eleven patients were included, of whom 168 received baricitinib and 243 received tocilizumab. Due to availability, baricitinib was used when the Delta and Omicron variants were dominant, and tocilizumab was used throughout the entire study period. There was no significant difference in percentage of patients with clinical improvement at day 14 after immunomodulator initiation (35% versus 36%, p=0.82). Progression to mechanical ventilation (MV) or ICU care occurred significantly less frequently in baricitinib recipients compared to tocilizumab recipients (47% versus 57%, p=0.04). No significant differences were observed for in-hospital mortality, clinical improvement at days 7 and 28 after immunomodulator initiation, and incidence of VTE.

**Conclusion:**

Baricitinib and tocilizumab appear to produce similar clinical outcomes in patients with severe or critical COVID-19. Baricitinib recipients had decreased progression to MV or ICU care, however, it remains unclear whether this outcome was influenced by changes in standard of care and circulating variants at different time points throughout the pandemic.

**Disclosures:**

**All Authors**: No reported disclosures

